# Physical exercise and college students’ sense of meaning in life: Chain mediating effect test

**DOI:** 10.1186/s40359-024-01792-9

**Published:** 2024-05-23

**Authors:** Hongbo Zhao, Xue Yin

**Affiliations:** School of Physical Education, Liaoning Normal University, Dalian, China

**Keywords:** Physical exercise, Self-control, Self-concept, A sense of meaning in life, College students

## Abstract

**Background:**

To explore the impact mechanism of physical exercise on the sense of meaning in life of college students, and analyze the chain mediating effect between self-concept and self-control.

**Methods:**

A questionnaire survey was conducted on 923 college students in China using the Physical Exercise Rating Scale (PARS-3), Life Meaning Scale, Self Concept Scale, and Self Control Scale.

**Results:**

(1) Physical exercise, self-concept, self-control and sense of meaning in life are significantly related to each other; (2) Self-concept and self-control play a chain mediating role between physical exercise and college students’ sense of meaning in life, with an effect value of 0.042, accounting for 5.48% of the total effect.

**Conclusion:**

Physical exercise can directly enhance the sense of meaning in life of college students, and can also indirectly enhance it not only through the mediating effect of self-concept and self-control, but also through the chain mediating effect of the two. These results provide theoretical reference for college students to improve their sense of life meaning and mental health education.

## Background

College students are the watchers of social development. College students are the inheritors of knowledge and skills, the active promoters of social change and progress, the future builders of the country and the backbone of development, and bear the heavy responsibility of national development [1]. However, under the pressure of the current fast-paced and competitive field, not all college students can be more and more courageous and indomitable. Some college students resist the gradually “involutionalization” social development mode by breaking the can and breaking the can, which is now called “pendulum” [2]. This way of doing things is now known as “swinging rotten“ [3]. Behind the gradual evolution of the Internet buzzword from “Buddha system“ [4] to “lying flat“ [5] and now “swinging rotten”, the seemingly open-minded and flirtatious language revelry is the emergence of the existential anxiety of some college students [6], which has resulted in the blurring of self-orientation and a sense of meaning in life [7]. Behind the seemingly open-minded linguistic revelry is a true portrayal of some college students who, under the existential anxiety, have a blurred self-positioning and a declining sense of the meaning in life, and are gradually falling into the abyss of nothingness [8]. The sense of meaning in life refers to the individual’s ability to perceive life, understand life, and realize the value, goal and mission of life [9]. Its predictive effect on negative emotions and coping styles and its moderating effect on stress and anxiety have been confirmed by many studies [10]. College students, as the main force of the young generation and an important training object of the strategy of developing the country with talents, should be stimulated by youth and burn their lives in struggle, but the data of the survey on the level of college students’ sense of the meaning in life are not optimistic according to the current research [11]. However, the data of the survey on the level of meaning of life of college students is not optimistic, and quite a number of college students do not know how to fight against the emptiness of life and live out the meaning of life through what way. From the perspective of physical education, the study has shown that physical exercise has a very high value of mental health [12]. However, the research on the role of physical exercise in college students’ sense of meaning in life and its mechanism of action is still insufficient, and there are still many areas to be explored. The present study aims to investigate the relationship between physical exercise and college students’ sense of meaning in life and the mechanism of its influence, with a view to providing theoretical references and bases for improving college students’ sense of meaning in life and guaranteeing the implementation of the strategy of developing the country by talents in the new era.

### The relationship between physical exercise and college students’ sense of meaning in life

As a proven psychological intervention, physical exercise is significant in improving mental health, and a sense of meaning in life, as a positive indicator of mental health, is deeply linked to physical exercise [13].McMann et al.‘s study showed that those who regularly participate in physical exercise have significantly more positive psychological states such as happiness and pleasure, and under such psychological variables, individuals tend to perceive more meaning in life [14] ; not only that, physical exercise also helps to increase the interaction and cooperation among college students and cultivate harmonious interpersonal relationships, which in turn promotes the formation of a sense of meaning [15]. Zhang has also confirmed that interpersonal relationships are significantly positively correlated with the sense of meaning in life [16]. Moreover, some studies at home and abroad have pointed out that physical exercise is significantly positively correlated with the sense of meaning in life among adolescents [17]. Physical exercise can also enhance the subjective well-being of older adults by improving their sense of meaning in life [18]. Based on this, the present study proposes hypothesis H1: Physical exercise positively predicts college students’ sense of meaning in life.

### Physical exercise, self-concept and college students’ sense of meaning in life

Self-concept is an individual’s self-awareness of various aspects of himself, including self-description and self-evaluation [19]. Self-concept is a positive predictor of college students’ mental health [20]and is closely related to the sense of meaning in life. The research points out that college students’ attitude towards themselves reflects whether they have the ability to evaluate objectively, and thus whether they can evaluate the value of life objectively [21]; College students’ understanding of their own life is influenced by the content related to self-evaluation [22]. The higher the level of self-concept of college students, the more inclined they are to perceive the meaning of life by satisfying the needs of autonomy and belonging [23]. Moreover, empirical studies have shown that self-concept clarity plays an important role in the structure of self-concept, and there is a significant correlation between it and college students’ experience of the sense of life meaning [24]. Optimism can indirectly affect life’s meaning by influencing middle school students’ self-concept [25]. In addition, the practice of physical exercise can have an essential role in the structure of self-concept. In addition, the practice of physical exercise can contribute to the level of physical self-concept by enhancing the experience of self-efficacy [26]. Another study has confirmed that middle-aged women can increase their self-concept by practicing physical exercise to enhance their evaluation of their bodies [27]; at the same time, physical intervention training can also influence the improvement of the self-concept of college students in higher vocational colleges and universities [28]. Based on this, the present study proposed the hypothesis H2: self-concept mediates the relationship between physical exercise and college students’ sense of meaning in life.

### Physical exercise, self-control, and college students’ sense of meaning in life

Self-control is the ability of an individual to transcend or change internal reactions consciously [29]; poor self-control may be a risk factor for negative emotions, and studies have shown that people with lower self-control are more likely to be depressed than those with higher self-control [30]. College students with higher levels of meaning in life can push themselves to make more positive and fewer negative changes; the meaning of life therapy is even more effective in allowing depressed patients to improve their symptoms of depression and anxiety [31]. It can be seen that there is a close relationship between a sense of meaning in life and self-control. A sense of purpose contains two dimensions: existence and meaning-seeking [32]. Research has shown that self-control fully mediates the relationship between representing existence and cell phone addiction tendency and partially mediates the relationship between meaning-seeking and addiction tendency [33]. Overseas studies have shown that individual trait self-control positively correlates with the sense of meaning in life; self-control has a significant role in the relationship between meaning in life and meaning in college students [34]. Self-control partially mediates and moderates the relationship between a sense of energy and depression among college students [35]. The self-control strength model emphasizes self-control’s importance in the relationship between reason and depression. In addition, the strength of the self-control model emphasizes that self-control has a limited reserve of resources and that positive emotional affect can compensate for the depletion of self-control. When college students do physical exercise, through physical participation and partner cooperation, they can effectively release physical and mental depression and negative emotions, gain positive emotional experience such as confidence, hope, optimism and friendship, and gradually improve self-control ability [36]. Physical exercise can help participants effectively release physical and mental suppression and negative emotions through physical participation and peer cooperation. It has been shown that there is a significant positive correlation between physical exercise and junior high school students’ self-control levels [37]. The story of physical exercise and the amount of physical exercise are related to self-control [38]. The amount of physical exercise is positively correlated with self-control, and the level of physical exercise and the amount of physical exercise is positively associated with self-control [39]. Based on this, the present study proposed hypothesis H3: self-control mediates the relationship between physical exercise and college students’ sense of meaning in life.

### Chain mediation assumption

Research has confirmed that there is a positive association between an individual’s increased level of self-concept and their ability to regulate and control perceptions and attitudes toward events and that low self-concept clarity inhibits self-control [40]; self-concept clarity and self-control mediate the relationship between negative affective states and emotional eating among Chinese adolescents [41]; level of explanation and cross-situational consistency of self-concept explained 17% of self-control, both of which were significant predictors [42]. Based on this, the present study proposed Hypothesis H4: Self-concept and self-control may mediate between physical exercise and college students’ sense of meaning in life.

### The current study

**Fig. 1:**
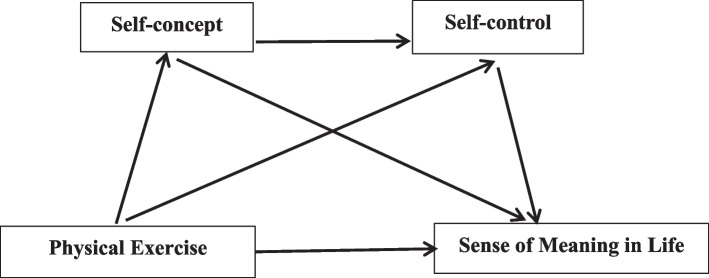
Hypothetical models of physical exercise, self-concept, self-control and sense of meaning in life

There is a close relationship between physical exercise, college students’ sense of meaning in life, self-concept, and self-control. Although relevant studies have confirmed some of the relationships among the four factors, no study has examined the mechanism of physical exercise, self-concept, and self-control combined to investigate their effects on college students’ sense of meaning in life. Therefore, this study aims to explore the internal influence mechanism of college students’ sense of meaning in life and analyze how physical exercise affects college students’ sense of meaning in life and the chain mediating role of self-concept and self-control in between. Based on previous theories and studies, the hypothesized model shown in Fig. 1 is constructed.

## Methods

### Participants

Taking college students’ physical exercise, self-concept, self-control, sense of life meaning and the relationship among various variables as the research objects, this study adopts stratified random sampling method, and takes freshmen to seniors from Dalian Maritime University and Liaoning Normal University and students of different majors as the investigation objects. The number of subjects in each dimension is relatively balanced, and the number of subjects is large. It can represent the real situation of each group relatively accurately. The basic information of the selected research objects in this study is shown in Table 1.

**Table 1:** Basic information of survey respondents (*N* = 923)

Demographic Variables	Categorization	Number of People	Percentage (%)
Gender	Men	354	38.4%
	Female	569	61.6%
Grade	First Year	326	35.3%
	Sophomore Year	200	21.7%
	Junior Year	192	20.8%
	Senior Year	205	22.2%
Specialized Field	Literature and History	194	21.0%
	Science and Engineering	518	56.1%
	Economic Management	132	14.3%
	Art and Physical Education	79	8.6%
Location of Their Home	Countryside	385	41.7%
	Municipalities	538	58.3%
Only Child	Yes	465	50.3%
	No	458	49.7%

### Research methodology

Questionnaire survey method was adopted in this study. An electronic questionnaire was made through the website of the questionnaire website. From April 8, 2023 to May 12, 2023, teachers of public courses, secretaries of colleges and students’ unions of colleges and universities sent the link of the electronic questionnaire to more than 1000 undergraduates of Dalian Maritime University and Liaoning Normal University. A total of 1015 questionnaires were collected, and after excluding invalid samples such as incomplete answers and regular answers, the final effective sample size was 923, with an effective rate of 91%. Specific survey and measurement tools are as follows:

###  Physical exercise measurements

This paper selects the Physical Activity Rating Scale (PARS-3) compiled by Japanese scholar Masao Hashimoto and revised by Chinese scholar Liang Deqing et al. [43].The Chinese version of PARS scale can better evaluate the use of physical exercise in general college students, including the intensity, frequency and time of physical activity.Assessment method: physical exercise = intensit×(time-1)×frequency. According to the criteria for rating the level of physical exercise, a score of ≤ 19 indicates a small level of physical exercise, a score between 20 and 42 indicates a medium level of physical exercise, and a score of ≥ 42 indicates a large level of physical exercise. The Cronbach’s alpha coefficient for the scale in this study was 0.779.

#### Measurement of sense of meaning in life

The Meaning of Life Questionnaire (MLQ) is divided into two dimensions: having a sense of meaning and seeking a sense of purpose. A 7-point scale is used, with higher scores indicating a greater sense of meaning in life for the individual.Xinqiang Wang (2013) found that the Chinese revised version of MLQ has good reliability and validity among Chinese college students. Therefore, this study adopts the life meaning questionnaire revised by Xinqiang Wang to conduct a questionnaire survey [44].The Cronbach’s alpha coefficient of the scale in this study was 0.788.

### Self-concept measures

Yang Xiaoyan (2002) first translated and used the Wallace Self-Concept Scale (WSCS), which operates 15 sets of adjectives with opposing concepts and a 7-point scale, with higher scores indicating that the individual views themself more positively. A reliability test determined its applicability to the Chinese student population, so the present study proposed to use the revised WSCS to test the scale [45]. The Cronbach’s alpha coefficient of the scale was 0.901.

### Self-control measurements

The Self-Control Scale (SCS), currently the most used in China and revised by Tan et al. (2008), was used [46]. The scale consists of 19 questions and contains five dimensions: impulse control, healthy habits, resisting temptation, focusing on work, and moderating entertainment. A 5-point scale is used, with higher scores indicating greater individual self-control. The Cronbach’s alpha coefficient for the scale in this study was 0.810.

### Statistical processing

1. SPSS 27.0 software was used to process and analyze the data, including descriptive statistics of physical exercise, sense of life meaning, self-concept and self-control, difference test (using independent sample t test and one-way ANOVA), correlation test and hierarchical regression test. The Harman single factor method was used to check for common method bias.
2. Model 6 in the SPSS macro program Process was used to test the mediating effects [47]. The main tests were: the direct relationship between physical exercise and a sense of meaning in life, the mediating role of self-concept and self-control, and the chain mediating role of physical exercise and a sense of meaning in life.
3. Bootstrap method was used to test whether self-concept and self-control had a chain-mediated effect between physical exercise and sense of meaning in life.

## Results

### Common method bias test

To test the possible common method bias in the questionnaire, six reverse scoring questions were designed in the self-concept scale and tested using Harman’s one-factor method, which showed that there were 11 common factors with eigenvalues > 1, of which the first factor explained 23.226% of the variance, which was less than the critical value of 40%, which shows that there is no standard severe method bias in this study.

### Tests of variance

The independent samples t-test was used to test the differences in physical exercise, sense of meaning in life, self-concept, and self-control of college students by place of birth, and the results showed that urban college students scored significantly higher than rural college students in physical exercise and sense of meaning in life, but self-concept and self-control did not differ considerably in the place of birth variable. A one-way ANOVA was used to test for differences in physical exercise, sense of meaning in life, self-concept, and self-control among college students regarding grade level and major. As shown in Table 2, there is no significant difference in physical exercise, sense of meaning in life, self-concept, and self-control of college students in terms of grade level.There is a significant difference in the physical exercise of college students in their majors (F = 3.11, *p* < 0.05), and there is no significant difference in the sense of meaning in life, self-concept, and self-control in their majors (Table 2).

**Table 2:** Tests for differences in the variables

Variant	Raw Floor	Grade	Specialty
Physical Exercise	2.479^*^	0.723	3.105^*^
Self-concept	1.757	1.156	0.459
Self-control	1.654	0.918	1.083
Sense of Meaning in Life	2.917^**^	1.902	0.123

Note: * *p* < 0.05,** *p* < 0.01,*** *p* < 0.001

Correlation analysis was used to analyze the relationship between the variables. The results showed that college students’ gender, physical exercise, sense of meaning in life, self-concept, and self-control were all correlated, but whether college students were only children or not were significantly correlated with physical exercise, self-concept, self-control, and sense of meaning in life (Table 3). The results of this analysis provide a sound basis for the subsequent mediation effect test, suggesting that self-concept and self-control can mediate between physical exercise and college students’ sense of meaning in life.

**Table 3:** Correlation analysis table

Variant	Gender	Only Child	Physical Exercise	Self-concept	Self-control	Sense of Meaning in Life
Gender	1					
Only Child	0.081*	1				
Physical Exercise	-0.039	-0.023	1			
Self-concept	0.036	-0.008	0.613***	1		
Self-control	-0.089**	-0.014	0.647***	0.468***	1	
Sense of Meaning in Life	-0.071*	-0.010	0.570***	0.464***	0.699***	1

Note: * *p* < 0.05,** *p* < 0.01,*** *p* < 0.001

Therefore, it is necessary to include birthplace, significance, and gender as control variables. As shown in Table 4, without controlling variables, it shows that physical exercise scores significantly and positively predicted the sense of meaning in life scores, β = 0.273, *p* < 0.001; while with controlling variables, physical exercise scores still particularly and positively predicted the importance of the definition of life scores,β = 0.273, *p* < 0.001, R2 = 0.329, F = 112.679, *p* < 0.001. This result suggests that the higher the level of physical exercise, the higher the sense of meaning in life, validating research hypothesis 1.

**Table 4:** Table of stratified regression results

Variant	Model 1		Model 2		Model 3		Model 4	
	Ratio	Demar- cate ratio	Ratio	Demar- cate ratio	Ratio	Demar- cate ratio	Ratio	Demar- cate ratio
Physical Exercise	0.276 ***	0.570	0.274 ***	0.565	0.274 ***	0.566	0.273 ***	0.564
Place of Origin Students			-0.829	-0.050	-0.839	-0.050	-0.847	-0.051
Specialized Field					0.097	0.010	-0.022	0.002
Gender							-0.838	-0.049
R2	0.324		0.327		0.327		0.329	
F	442.185***		223.327***		148.787***		112.679***	
∆R2	0.324		0.002		0.000		0.002	
∆F	442.185		3.344		0.130		3.259	

Note: * *p* < 0.05,** *p* < 0.01,*** *p* < 0.001

### Chain mediation model test

**Fig. 2:**
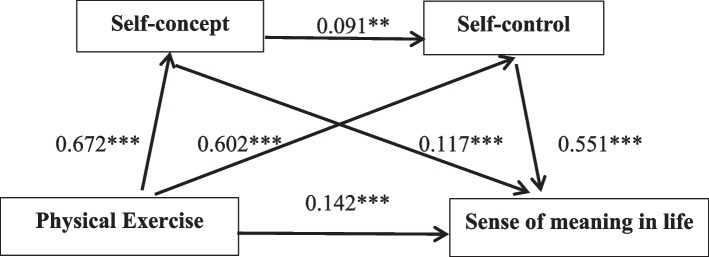
Model diagram of the mediation of self-concept and self-control between physical exercise and sense of meaning in life

Model No. 6 in the SPSS macro program plug-in Process prepared by Hayes was used for 5000 repetitive samplings. In this model, physical exercise was considered an independent variable, and a sense of meaning in life was a dependent variable. In contrast, self-concept and self-control were considered chained mediating variables. The results of the path coefficients are shown in Fig. 2. Significant results were obtained for the overall regression equation with an R-squared value of 0.335 and an F-value of 463.662 with a p-value of less than 0.001 (Table 5 for details).

**Table 5:** Regression analyses of chain mediation models of self-concept, self-control(standardized)

Variant		Physical Exercise	Self- concept	Self- control	R2	F
Self-concept	β	0.627			0.393	597.385***
	SE	0.034				
	t	24.442***				
Self-control	β	0.602	0.091		0.440	360.656***
	SE	0.022	0.017			
	t	19.007***	2.856**			
Sense of Meaning in Life	β	0.142	0.117	0.551	0.522	334.263***
	SE	0.046	0.030	0.059		
	t	4.117***	3.969***	18.081***		
Aggregate Effect	β	0.579			0.335	463.662***
	SE	0.036				
	t	21.533***				

Note: *p < 0.05,**p < 0.01,***p < 0.001

The total effect of physical exercise on the sense of meaning in life was 0.767 after testing the mediating product using the Bootstrap sampling method. Further analysis showed (Table 6) three significant mediating effects, proving hypotheses H2, H3, and H4. Firstly, the indirect impact produced by the path of “Physical exercise→Self-concept→Sense of meaning in life” was 0.097, accounting for 12.65% of the total effect, and the Bootstrap confidence interval did not contain 0. First, the indirect effect through the path of “physical exercise→self-concept→sense of meaning in life” is 0.097, accounting for 12.65% of the total impact, and the Bootstrap confidence interval does not contain 0, which indicates that “self-concept” plays a vital role in the relationship between “physical exercise” and “emotional stability.” This demonstrates that “self-concept” plays a significant mediating role between “physical exercise” and “emotional stability.” Secondly, the indirect effect through the path of “physical exercise→self-control→sense of meaning in life” is 0.440, accounting for 57.37% of the total impact, and the Bootstrap confidence interval does not contain 0, indicating that “self-control” plays a significant role in the relationship between “physical exercise” and “emotional stability.” The Bootstrap confidence interval does not contain 0, indicating that “self-control” plays a significant mediating role between “physical exercise” and “emotional stability.” Similarly, “self-concept” and “self-control” have a significant chain mediation between “physical exercise” and “emotional stability.” There is a considerable chain mediation between “physical exercise” and “emotional stability.” In conclusion, the above results indicate that physical exercise significantly affects sense of meaning in life through the mediating variables “self-concept” and “self-control.”

**Table 6:** Chained mediation model effect test for self-concept, self-control

Type of Benefit	Efficiency Value	Se	Bootstrap95%CI		Percentage of Relative Effects
			Lower Limit	Limit	
Aggregate Effect	0.767	0.036	0.697	0.837	100%
Direct Effect	0.189	0.046	0.099	0.279	24.64%
Physical Exercise → Self-concept → Sense of Meaning in Life	0.097	0.046	0.042	0.158	12.65%
Physical Exercise → Self-control → Sense of Meaning in Life	0.440	0.044	0.356	0.528	57.37%
Physical Exercise → Self-concept → Self-control → Sense of Meaning in Life	0.042	0.020	0.004	0.081	5.48%
Total Indirect Efect	0.579	0.046	0.490	0.672	75.49%

## Discussion

### The relationship between physical exercise and college students’ sense of meaning in life

This study shows a significant positive correlation between physical exercise and college students’ sense of meaning in life scores (*r* = 0.570). The positive predictive effect of physical exercise scores on the sense of purpose of life scores is significant with the inclusion of control and mediator variables (*p* < 0.001), which suggests that performing physical exercise is an effective way for college students to enhance their sense of meaning in life, which is in line with the results of the studies conducted by other scholars [48–50], and the research H1 hypothesis was verified. Many contemporary college students find it difficult to adapt to the rapidly changing social environment, coupled with the influence of the virtual world, growing pains and social comparison, and other reasons, there are problems such as confused goals, excessive pressure, negative life.The sense of meaning in life has a spiritual leading effect on college students to improve learning motivation and promote upward development. It belongs to the category of positive emotional experience that helps to enhance college students’ sense of well-being in life and reduce the risk of suicidal behaviors, which is of great significance to college students’ academic careers. By participating in physical exercise, college students can enhance their physical fitness, improve their endurance and strength, strengthen their immune systems, and help prevent diseases. Physical health and vitality are the basis for acquiring a sense of meaning in life. Physical exercise also helps to release pressure, relieve emotions, improve psychological quality, and cultivate an optimistic mindset, thus enabling college students to face life more positively and optimistically and seek life’s meaning and goals. The reason why the meaning of life is formed is inseparable from the satisfaction of basic psychological needs such as the need for autonomy, the need for competence, and the need for relationships [51]. And physical exercise satisfies the need for competence through constantly challenging the self and breaking through the self; college students can establish cooperative consciousness and team spirit in the process of sports, which can realize the need of relationship; and adjusts the activities of the sympathetic and parasympathetic nervous system by promoting the synthesis of 5-hydroxytryptamine and dopamine, increasing the secretion of endorphins [52], college students’ cognitive and conscious behaviors can maintain a high level of autonomy, realize that there are clear goals and missions in life, and discover the meaning and value of life, thus significantly enhancing the life satisfaction and physical and mental pleasure of college students, and genuinely experiencing a sense of meaning in life.

### Mediating relationship between self-concept and self-control among college students

Taking self-concept and self-control as mediating variables, this study built a mediating model of the impact of physical exercise on college students’ sense of life meaning. Among them, the mediating effect with self-concept as the mediating variable was 95%CI=[0.042,0.158], and the mediating effect with self-control as the mediating variable was 95%CI=[0.356,0.528]. All of them reached significant level.

### The mediating role of the self-concept

This study confirms that self-concept mediates the relationship between physical exercise and college students’ sense of meaning in life. College years are critical in forming and developing individual self-concepts, and college students are susceptible to various factors because of their unsettled thoughts. Physical exercise can influence self-concept by changing both internal and external aspects of college students, which is an essential factor in the development of self-concept. From the internal point of view, self-concept is a kind of cognition of the self. It has been shown that strength training can improve self-concept, and and college students’ active physical exercise can improve and promote cognitive function [53], a more rational and objective understanding of the self; from the external point of view, the fitness function of physical exercise can improve college students’ physical health beauty, body beauty, contour beauty, posture beauty and movement beauty, so that they can enhance their self-confidence in daily life and graduation job hunting, and improve their own evaluation through the sense of physical self-esteem. Their evaluation of themselves is improved through the sense acquisition of physical self-esteem. Moreover, the positive or negative cognitive evaluation of college students’ perceptions of themselves, i.e., whether their self-concept is skewed positive or not, tends to affect their views and perceptions of their interpersonal relationships with the environment. It has been pointed out that self-identity, as an aspect of self-concept, can positively predict the sense of meaning in life [54] and that self-concept clarity, as a crucial part of self-concept structure, also has a significant effect on the sense of meaning in life [55], and that the study of Ji and Liu directly pointed out that there is a significant positive correlation between college students’ self-concept and the sense of meaning in life, and that self-concept has a significant positive correlation with the sense of life [21]. Significant positive correlation between self-concept and a sense of meaning in life, and that self-concept can significantly predict college students’ sense of meaning in life. It is not difficult to conclude that physical exercise can change the physical and mental state of college students to promote a positive self-concept, which is conducive to interpersonal communication and environmental adaptation, and thus perceive more meaning in life [56].

### The mediating role of self-control

This study confirms that self-control mediates the relationship between physical exercise and college students’ sense of meaning in life. Tao et al. indicated that physical exercise can help college students enhance attentional control [57]. First of all, Physical exercise can promote the development and functional strengthening of college students’ nervous system, improve the synthesis and release of neurotransmitters, enhance nerve conduction speed, improve neuromuscular coordination, prevent nervous system diseases, and promote nerve regeneration and recovery, and the development of the nervous system directly affects the formation and development of college students’ self-control ability [58]. Secondly, by participating in physical exercise, college students can develop and improve their willpower and overall self-control ability [59]. Willpower is an college students’ ability to pursue goals and overcome difficulties and temptations. In the process of physical exercise, college students need to maintain sustained dedication and perseverance to cope with a certain degree of physical load and psychological challenges, and this process exercises and strengthens the college students’ willpower. Such training will help college students to better manage their desires and impulses in other areas of life, adhere to a task more consistently, and maintain stable behavioral patterns [52]. In addition, emotional experiences play a role in self-control, and adverse emotional experiences can lead to a sense of loss of control [59]. Negative emotional experiences can lead to feeling “out of control.“Physical exercise can release neurotransmitters such as endorphins and dopamine in the body, improve the happiness and emotional stability of college students, and when college students are emotionally stable, it is easier to maintain self-control. Self-control positively predicts college students’ sense of meaning in life. First, self-control helps college students develop positive behavioral habits and a healthy lifestyle [60]. Through self-control, college students can plan their study and recreation breaks and insist on fitness, and these positive behavioral habits benefit their physical and mental health and enhance their sense of meaning and happiness; second, self-control helps college students overcome setbacks and difficulties. College students may face challenges, failures, and pressures in college, such as unsatisfactory exams, blocked research projects, and interpersonal relationship problems. Through self-control, college students can remain calm and collected, actively seek ways and strategies to solve problems, and persist in their efforts to pursue their goals. This persistence and perseverance will enhance their self-confidence and sense of achievement and enrich their sense of meaning in life; self-control helps college students establish positive interpersonal relationships [61]. Through self-control, college students can control their emotions and behaviors and show friendly, respectful, and cooperative attitudes in their interactions. Positive interpersonal relationships will provide them with support, understanding, and a sense of security and enhance their sense of meaning in life and mind of belonging. Therefore, college students should strengthen the exercise of self-control ability.

### Chain mediators of physical exercise’s influence on college students’ sense of meaning in life

The Bootstrap method verified the chain-mediated roles of self-concept and self-control in the positive effect of physical exercise on college students’ sense of meaning in life. The constructed chain mediation model provides a new perspective for further advancing and understanding the relationship between physical exercise and a college student’s sense of purpose in life in the future. First, physical exercise promotes cognitive development, affecting the sense of meaning in life. Self-concept is a perception of the self, and research has found that those individuals with a positive self-concept are more likely to feel a sense of meaning and purpose in life. A positive self-concept includes self-esteem, self-confidence, and a sense of self-actualization, which make individuals more motivated to pursue a meaningful life [62]. Their affirmation and trust in their worth make it easier for them to find meaning in their lives. Cognition is a bodily experience, and no cognition exists without the body [63]. Physical health and a sound mind are the basis for college students to perceive the meaning of life, and physical exercise is an essential channel for developing both. In addition, one of the protective factors of self-control is the ability of self-monitoring and self-reflection in the self-concept, through which one can evaluate one’s behavior and implementation to regulate and control one’s impulses and desires, which can help college students to carry out interpersonal interactions and environmental adaptation better, and indirectly perceive more meaning of life [56]. Secondly, physical exercise can improve the individual’s executive function and self-management ability and promote the development of self-control [64]. High self-control is associated with stronger goal orientation, self-discipline, and a sense of power to realize [64], which can enhance the enduring pursuit and attainment of personal goals and the importance of purpose and meaning in life. Therefore, college students should be encouraged to find themselves, mold themselves, and control themselves through physical exercise, thus giving more meaning and value to their lives.

## Conclusion

Physical exercise can directly increase college students’ sense of meaning in life and indirectly through the mediating role of self-concept and self-control and the chain mediating part of both.Therefore, in the training process of college students, first of all, colleges and universities should pay attention to the impact of the sense of meaning of life on the psychology of college students, and make the sense of meaning of life of college students accept intervention and change. Secondly, pay attention to the regulating effects of physical exercise, self-concept and self-control on the sense of life meaning of college students.

### Limitations and future prospects

This study explores the relationship between perceived physical exercise and college students’ sense of meaning in life. Constructing a chain mediation model reveals the inner mechanism of the influence of physical exercise on college students’ sense of meaning in life, which has significant theoretical and practical value for understanding college students’ sense of meaning in life. Also, it provides a prerequisite for further research on improving college students’ sense of meaning in life. However, this study needs to be further improved: First, the results of this study are only limited to the mediating role of self-concept and self-control in physical exercise and college students’ sense of meaning in life. Other mediating variables, such as social support, mental toughness, and parenting styles, need to be further explored in subsequent studies. Second, universities include junior college students, undergraduates and graduate students. At the same time, the study population is students from first-year students to seniors, the sample source is relatively single, and caution is needed when variables such as physical exercise and sense of meaning in life are extended to other academic segments. Future studies need to expand the sample to obtain a higher level of representativeness. Third, this study used a questionnaire survey of college students; however, it is essential to note that the results may be limited due to the effects of common methodological bias that may be present in this approach. To explain the relationships between variables more fully, future studies may consider obtaining data from multiple sources, including teachers, peers, and parents, to obtain more accurate findings.

## Data Availability

The data presented in this study are available on request from the corresponding author.
